# The onset of acute type A aortic dissection following recovery of type B intramural haematoma: a case report

**DOI:** 10.1186/s12872-020-01440-1

**Published:** 2020-04-06

**Authors:** Kai Zhang, Song-Bo Dong, Xu-Dong Pan, Li-Zhong Sun

**Affiliations:** Department of Cardiovascular Surgery, Beijing Anzhen Hospital, Capital Medical University, Beijing Institute of Heart, Lung and Blood Vessel Diseases, 2 Anzhen Rd, Chaoyang District, Beijing, 100029 China

**Keywords:** Aortic intramural haematoma, Aortic dissection, Recurrence

## Abstract

**Background:**

Aortic intramural hematoma is a life-threatening condition reported with increasing frequency. It can be classified into Stanford type A or B depending on whether the ascending or descending aorta are involved, respectively. However, the onset of acute type A aortic dissection following recovery of type B intramural haematoma is rarely reported.

**Case presentation:**

We present an uncommon case of acute Stanford type A aortic dissection developing 3 months after recovery of type B IMH in a 47-year-old female.

She complained acute chest pain. The operation was successfully done. She was in good condition and asymptomatic at a 3-month follow-up.

**Conclusions:**

Type B intramural haematoma can lead to type A aortic dissection even after totally absorbed and the primary entry has the potential to be located in the ascending aorta. Unsatisfied blood pressure control may be the underlying cause.

## Background

Aortic intramural haematoma (IMH) is a form of acute aortic syndrome, accompanied by severe chest or back pain [1–3]. Aortic wall media hemorrhage without any intimal tear and a false aortic lumen defines IMH [2]. The diagnostic hallmark consist of a round or crescent-like region of aortic wall thickening ≥5 mm in size without blood flow upon computed tomographic angiography (CTA) or magnetic resonance imaging (MRI). Similar to classic aortic dissection, IMH can be classified into Stanford type A or B depending on whether the ascending or descending aorta are involved, respectively. Type B IMH is more common (60–70%) [1, 3]. Optimal management is still controversial as aortic IMH patient courses can vary considerably [4]. Some patients recover under medical therapy, while others progress to aortic dissection requiring urgent surgical treatment. Early operation is recommended particularly in the case of a type A (involving the ascending aorta) IMH [5, 6]. In this report, we present a case of acute Stanford type A aortic dissection developing 3 months after recovery of type B IMH, including medical therapy, surgical strategy, postoperative management, and experience summary.

## Case presentation

**Fig. 1:**
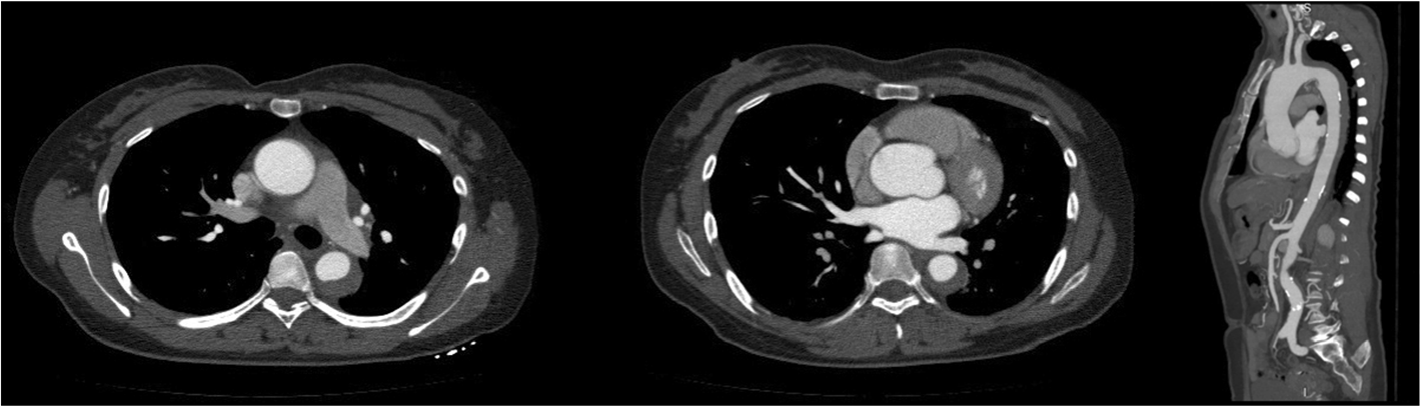
Initial chest CTA showing intramural hematoma around proximal part of the descending thoracic aorta

**Fig. 2:**
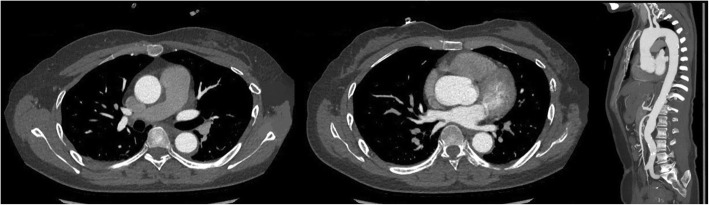
Complete resolution of intramural hematoma,1 month later

**Fig. 3:**
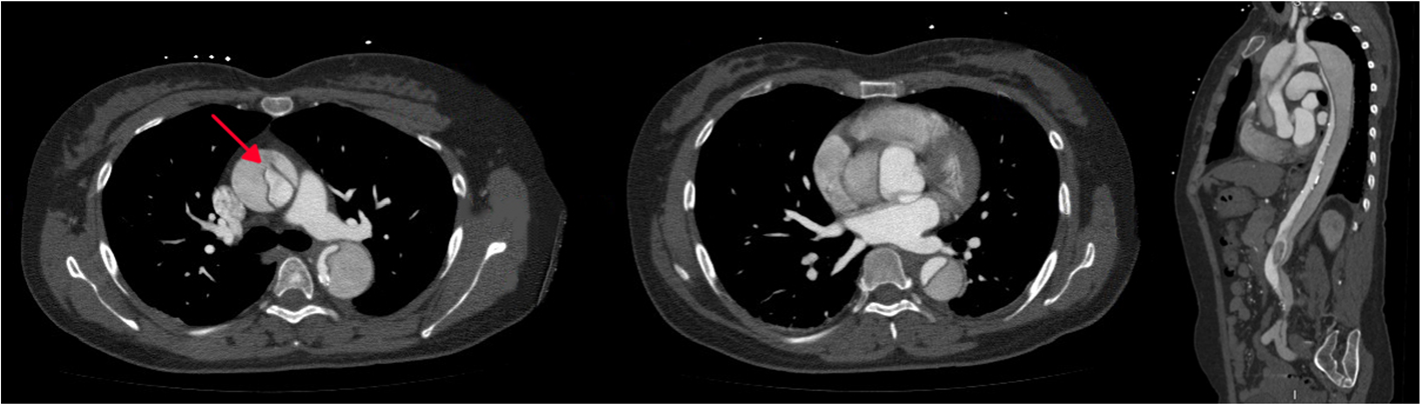
Progressing into aortic dissection, 3 months later. The red arrow points to the intimal tear

A 47-year-old female complaining of acute chest pain for 1 day, was admitted to the emergency room of our hospital on November 26, 2016. She had no history of hypertension but her blood pressure was 180/90 mmHg. She was never definitely diagnosed with type 2 diabetes mellitus or other diseases. She had no signs of secondary hypertension (including renal disorder, macroangiopathy, pregnancy-induced hypertension, chromaffinoma, primary hyperaldosteronism). Her family had no history of acute aortic syndrome or suspicion of any connective tissue disease, such as Marfan syndrome. Acute aortic syndrome was suspected based upon symptoms and course. A CTA scan revealed a Stanford type B aortic IMH involving the descending aorta from the left subclavian artery to the level of the diaphragm (Fig. 1). The diameter of the descending aorta was about 3.3 cm (including 1.3 cm crescent-like thickening at the level of the pulmonary artery bifurcation). The ascending aorta was not involved, and penetrating atherosclerotic ulcers was not found in the entire aorta. Emergency medicine specialist immediately gave the patient medical treatment, including analgesic treatment, tight blood pressure control and rehydration therapy. The follow-up CTA images marked decrease in the thickness of IMH and the symptoms of the patient also distinctly improved during hospitalization. After 2 weeks observation in our hospital, she was discharged home. On the follow-up CTA scan 1 month after the initial event, the IMH was thoroughly absorbed (The diameter of the descending aorta was still 3.3 cm at the level of the pulmonary artery bifurcation) and the aorta looked completely normal. (Fig. 2). Although she had been taking metoprolol and nifedipine, her blood pressure was hard to control, at approximately 160/90 mmHg during a resting state. She was transferred to our hospital again presenting with unrelieved chest pain 3 months later. After admission, a repeat CTA scan revealed an acute complex type A aortic dissection, involving the ascending aorta, aortic arch, and descending aorta (from the left subclavian artery to the left common iliac artery) (Fig. 3). An urgent bedside echocardiography was performed and showed acceptable cardiac function with a left ventricular ejection fraction of 60% and mild mitral regurgitation.

**Fig. 4:**
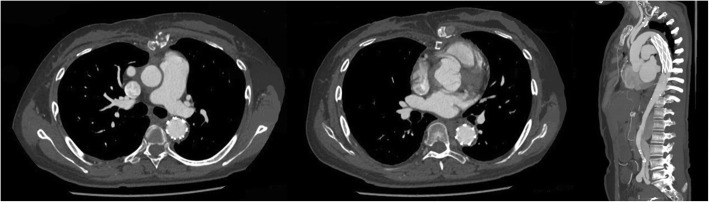
Postoperative re-examination, 3 months later

Emergency surgery was performed under the support of total cardiopulmonary bypass with selective cerebral perfusion via right axillary artery cannulation. We used an anterior approach via median sternotomy in order to manipulate the ascending aorta, where the risk of rupture was the highest. It was increased to 4.5 cm in diameter, with a dark red color over its length. Following induction of moderate hypothermic circulatory arrest, it was opened when the nasopharyngeal temperature reached 25 °C. The site of dissective entry was ~ 4 mm in size and located in the anterior wall intima in close proximity to the sinotubular junction. Based on the condition that Innominate artery, left common carotid artery and left subclavian artery were severe involved and a comprehensive review of the patient’s good physical condition, ascending aorta replacement and total arch replacement (TAR) with a frozen elephant trunk implant (FET) was implemented. After surgery, the patient underwent treatment with extensive debridement and continuous mediastinal irrigation because of a mediastinal infection. Fortunately, she survived and was discharged 1 month later. Pathology revealed the mucoid degeneration of the stoma and the broken and necrotic elastic fibers of the media. The patient was doing well and without new complaints at a 3-month follow-up (Fig. 4 and Supplementary Material: Fig. 5).

## Discussion

Risk factors for acute IMH are associated with increased wall stress and/or aortic media abnormalities [7]. The most common predisposing factor that cause increased wall stress is hypertension. It is observed in 65–75% of individuals and the majority of them are poorly controlled. Other factors associated with increased aortic wall stress include cocaine, chromaffinoma, weightlifting, physical trauma, and coarctation of the aorta. There are also a lot of conditions related to aortic media abnormalities, include pre-existing aortic valve disease, family history of aortic diseases, history of cardiac surgery, inflammatory vasculitis, atherosclerosis, and pregnancy. However, up-to-date data on distinctive predisposing factors between type A and type B IMH remain scarce, requiring further investigations.

How acute IMH progresses remains variable: some cases regress spontaneously, while others have unfavourable outcomes as in our case. IMH can lead to acute aortic dissection in 3–14% and 88% of patients suffering from type B and A IMH, respectively [1, 3]. Despite the generation of several hypotheses, the exact basis for aortic IMH remains uncertain [8, 9]. Spontaneous vasa vasorum rupture was originally proposed to underlie this condition [10], more recent work based on diagnostic imaging results suggests that many IMH patients exhibit intimal tears. As such, certain researchers propose that IMH be defined as acute aortic dissection without aortic false lumen flow [8, 11].

Acute IMH is a life-threatening condition requiring rapid medical therapy to reduce the risk of progressing to aortic dissection or rupture. Metoprolol (beta-blockers), morphine, and sodium nitroprusside represent first-line drugs. We should determine how best to manage IMH after initial treatment. General consensus supports early surgery for type A IMH patients because medical therapy in these cases results in limited effectiveness. However, the case for type B IMH is much more different. According to a latest literature [7], type B is divided into complicated type and uncomplicated type. Uncomplicated type B IMH has lower rates of adverse outcomes, with < 10% in-hospital mortality rates, leading to the recommendation that patients undergo initial conservative therapy with MRI or CTA-based surveillance. In complicated type B IMH, surgical repair should be advocated; Endovascular treatment is recommended if there are favorable anatomical structures and appropriate vascular access.

This case is rare. The ascending aorta was intact on the first CTA scan and still normal on the follow-up CTA scan 1 month later. However, a repeat CTA scan revealed the primary entry was located in the ascending aorta when the patient returned to our hospital again. It is reasonable to attribute the sudden deterioration to unsatisfied blood pressure control and a recent history of IMH since she had no family history of any connective tissue disease. In a recent study [11], up to 70% of type B IMH patients had a history of hypertension. However, the awareness rate and control rate of hypertension were inadequate, with 26% not undergoing anti-hypertensive therapy prior to hospitalization. Following discharge, 53% had high blood pressure. Furthermore, 60% went on to suffer from aortic aneurysm by the mid-term follow up. This clearly demonstrates that it is important to control blood pressure in order to prevent IMH-associated complications. In addition, although the patient’s IMH had been absorbed and the primary entry was located in the ascending aorta, the aortic wall was already weakening and was easily affected by hypertension, leading to the formation of the classic intimal flap of aortic dissection. There were several advantages to using TAR with FET for this patient. The intimal tear was sealed following FET implantation in the area reached by the surgical stent graft, and the distal aortic arch and proximal descending aorta were stabilized. More importantly, the use of this technique led to true lumen enlargement, re-established flow in both the true lumen and the side branches, promoted residual dissected aorta thrombosis, facilitated dissected aortic wall remodelling, and mediated aorta wall shrinkage [12].

What we must take away from this case is that type B IMH can lead to type A aortic dissection even after totally absorbed, and the primary entry has the potential to be located in the ascending aorta. Second, blood pressure management is also one of the cornerstones of prevention. Furthermore, when faced with this type of patient, it is important to determine whether surgery is indicated quickly. Last but not least, careful follow-up is essential for a patient with IMH.

## Supplementary information

**Additional file 1: Fig. 5.** Three dimensional reconstructions at: (A) Initial intramural hematoma, (B) Complete resolution of intramural hematoma, (C) Progressing into aortic dissection, (D) Postoperative re-examination.

## Data Availability

All data can be found at the Department of Cardiovascular Surgery, Beijing Anzhen Hospital.

## Abbreviations

IMH: Intramural haematoma
CTA: Computed tomographic angiography
MRI: Magnetic resonance imaging
TAR: Total arch replacement
FET: A frozen elephant trunk implant
