# Supplementary figures and images for: The onset of acute type A aortic dissection following recovery of type B intramural haematoma: a case report
Source: BMC Cardiovasc Disord. 2020 Apr 6;20:162. doi: 10.1186/s12872-020-01440-1 (PMC7137196; doi:10.1186/s12872-020-01440-1)

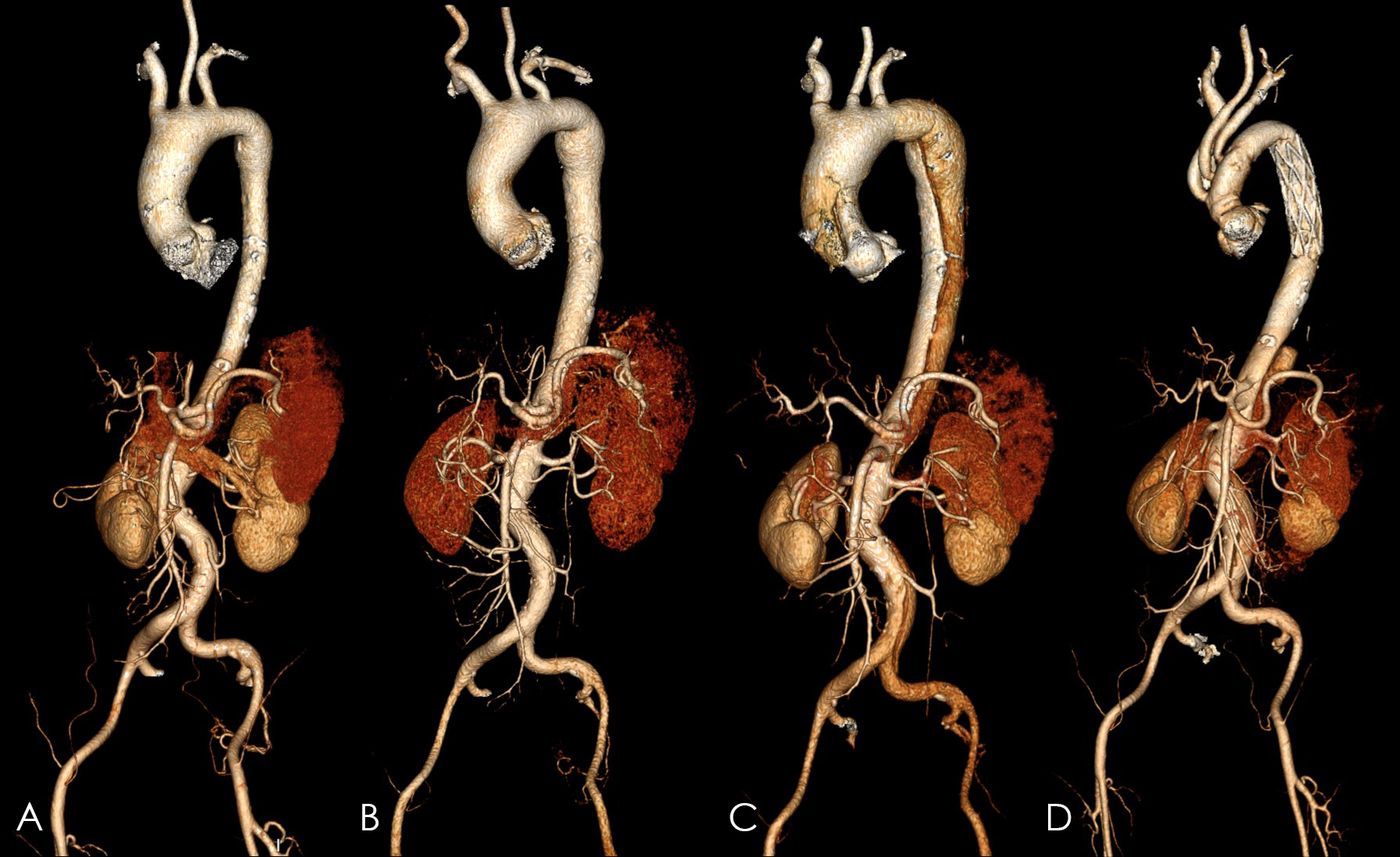

Supplement: Supplementary file 1 — Additional file 1: Fig. 5. Three dimensional reconstructions at: (A) Initial intramural hematoma, (B) Complete resolution of intramural hematoma, (C) Progressing into aortic dissection, (D) Postoperative re-examination. [file 12872_2020_1440_MOESM1_ESM.jpg]
